# Cognitive impairment in Parkinson's Disease using Telephone Montreal Cognitive Assessment version: Preliminary results

**DOI:** 10.1002/alz70857_107481

**Published:** 2025-12-26

**Authors:** Sheila Castro‐Suarez, Jonathan Adrian Zegarra‐Valdivia, Erik Alberto Guevara‐Silva, Cintia Margoth Armas‐Puente, Jeremy Esquivel‐Luna, Mario Cornejo‐Olivas

**Affiliations:** ^1^ Global Brain Health Institute (GBHI), University of California San Francisco, San Francisco, CA, USA; ^2^ CBI en Demencias y Enfermedades Desmielinizantes del Sistema Nervioso, Instituto Nacional de Ciencias Neurológicas, Lima, Peru; ^3^ Universidad Señor de Sipán, Chiclayo, Peru; ^4^ Instituto Nacional de Ciencias Neurologicas, Lima, Peru; ^5^ Hospital San Juan de Lurigancho, Lima, Lima, Peru; ^6^ Neurogenetics Working Group, Universidad Cientifica del Sur, Lima, Peru; ^7^ Neurogenetics Research Center, Instituto Nacional de Ciencias Neurológicas, Lima, Peru

## Abstract

**Background:**

Mild cognitive impairment (MCI) and dementia are non‐motor symptoms of Parkinson's disease (PD). The Telephone Montreal Cognitive Assessment (T‐MoCA) is a quick tool designed to screen for cognitive dysfunction remotely; it allows for clinical decision‐making.

**Method:**

Cross‐sectional study carried out during 2022‐2024 in Lima Peru. Eligible subjects were screened remotely by phone using T‐MoCA, Geriatric depression scale (GDS) and, and Pfeffer functional activities questionnaire. The T‐MoCA scores for each group were: cognitively unimpaired (18‐22), MCI (15‐17) and dementia (<15). Groups were compared using ANOVA, and a Spearman correlation matrix was used to explore relationships between sociodemographic and cognitive variables. Ethical approval was obtained by CIEI‐INCN.

**Result:**

We screened 155 PD cases, 60.7% men. Age at onset were 57.6 ±13.8, age at examination were 63± 12.9. The average of year of education of 11 [6‐14]. We divided the patients into 3 groups: cognitively unimpaired (*n* = 42), MCI (*n* = 40), and dementia (*n* = 73); and the T‐MoCA average score was 19.4± 1.3, 16.1 ± 0.74, and 10.1 ±3.14, respectively. No differences were found between the groups by sex. The GDS average score was 6.6±3.8 points suggesting mild depression. Years of education positively correlated with sentences repetition, abstraction, and the total T‐MoCA score (*r* = 0.56, r=0.63 and r = 0.65, respectively). The Pfeffer Score showed a negative correlation with the T‐MoCA (*r* = ‐0.54), reflecting an increase in functional dependence in patients with greater impairment.

**Conclusion:**

The T‐MoCA is a remote tool able to differentiate degrees of cognitive impairment in PD and emphasize the importance of sociodemographic factors, such as years of education, in cognitive performance.

